# Metagenomics-based study of rhizospheric microorganisms of *Poa alpigena L.* in Qinghai Lake, Ganzi River Plateau

**DOI:** 10.3389/fpls.2024.1518637

**Published:** 2025-02-21

**Authors:** Bo Wei, Qianqian Xu, Junfei Kong, Xu Su, Kelong Chen, Hengsheng Wang

**Affiliations:** ^1^ School of Biology, Food and Environment, Hefei University, Hefei, Anhui, China; ^2^ National Engineering Laboratory of Crop Stress Resistance Breeding, Anhui Agricultural University, Hefei, Anhui, China; ^3^ Anhui Promotion Center for Technology Achievements Transfer, Anhui Academy of Science and Technology, Hefei, Anhui, China; ^4^ Key Laboratory of Biodiversity Formation Mechanism and Comprehensive Utilization of the Qinghai-Tibet Plateau in Qinghai Province, Qinghai Normal University, Xining, Qinghai, China; ^5^ College of Geographic Sciences, Qinghai Normal University, Xining, Qinghai, China; ^6^ School of Biological and Food Engineering, Hefei Normal University, Hefei, Anhui, China

**Keywords:** Ganzi River, *Poa alpigena* L., microorganism, metagenomic sequencing, rhizosphere soil

## Abstract

**Introduction:**

*Poa alpigena* Lindm., a dominant forage grass on the Tibetan Plateau, plays a critical role in livestock production and grassland restoration. This study investigates the rhizospheric and non-rhizospheric soil microorganisms of *Poa alpigena L.* in the Ganzi River area of the Qinghai Lake basin using metagenomic sequencing to understand their diversity and potential ecological functions.

**Methods:**

Soil samples were collected from rhizospheric and non-rhizospheric areas of *Poa alpigena L.* using the S-type five-point sampling method. DNA was extracted, and metagenomic sequencing was performed using the BGISEQ-500 platform. Alpha and Beta diversity analyses were conducted, and LEfSe analysis was used to identify differentially abundant microbial taxa and metabolic pathways.

**Results:**

A total of 5,681 microbial species across 1,606 genera, 521 families, 61 phyla, and 246 orders were identified. Non-rhizospheric soils exhibited higher species richness than rhizospheric soils. Proteobacteria was the most abundant phylum in both soil types. Rhizospheric soils showed significant enrichment in pathways related to antibiotic biosynthesis, carbon metabolism, and methane metabolism, while non-rhizospheric soils were enriched in quorum sensing and drug-metabolizing pathways.

**Discussion:**

The findings highlight the selective influence of *Poa alpigena L.* on soil microbial communities and their potential role in mitigating methane emissions. The study provides a foundation for understanding the ecological functions of soil microorganisms in alpine meadows and supports sustainable grassland management practices.

## 1 Introduction

Soil microorganisms, mainly composed of soil bacteria, archaea, and fungi, are widely distributed in rhizosphere and non-rhizosphere soils. Their diversity and abundance play a key role in regulating ecosystem functions such as organic matter decomposition, plant productivity, soil carbon dynamics, nutrient cycling, and organic matter decomposition (Cheng et al., 2022; Yang et al., 2023). As a medium for material exchange between plants and soil ecosystems, rhizospheric microorganisms are essential for plant growth and development, enhancing plant productivity and resilience, regulating the biogeochemical cycling of biogenic elements in rhizosphere sediments, and can promote plant growth by regulating the ecology of rhizospheric microorganisms (Xie et al., 2021; Rehan et al., 2023).

The Qinghai Lake basin is a natural barrier in maintaining ecological security in the northeastern part of the Tibetan Plateau. It stops the spread of desertification toward the east in the western part of the plateau and is an important source of water vapor and climate regulation. It is also one of the more important areas rich in germplasm and biodiversity and has been included in a list of internationally important wetlands (Jia et al., 2021). The unique habitat of the Qinghai Lake area may harbor rich soil microbial resources. The region is sparsely populated and plays a minor role in China’s economic development, but its ecological function is of great significance (Wang et al., 2022). However, overgrazing and exploitation have degraded alpine meadows in the region to varying degrees. The restoration of alpine meadows and ecological conservation have become a concern for ecologists worldwide (Wang et al., 2020). Studies have shown that the degradation of alpine meadows is mainly caused by an imbalance in energy flows and material cycling in the ecosystem (Chu et al., 2020). The rhizosphere is the site of soil-plant-microorganism interactions and the hub of material cycling and energy exchange. It is important, not only for nutrient transformation in the soil, but also exerts a strong influence on the activity of rhizospheric microorganisms (Naeem et al., 2022). Therefore, exploring the diversity of rhizospheric and non-rhizosphere microorganisms and their characteristics are important for studying the cycling of soil nutrients during alpine meadow degradation.

*Poa alpigena* L. is an important forage grass of alpine meadows in the Qinghai Lake region. Being drought tolerant and cold resistant, it is one of the dominant forage grasses in the region (Dong et al., 2020; Shi et al., 2022). The study of rhizospheric microbial communities of *P. alpigena* L. is important for the conservation, desertification management, and ecological restoration of alpine grasslands (Dong et al., 2021). In this paper, we employed metagenomic and bioinformatic approaches to analyze differences between rhizospheric and non-rhizosphere microorganisms of *P. alpigena* L. in the Ganzi River area of Qinghai Lake. Our objective was to elucidate the structural changes in microbial communities and the main soil physicochemical factors, in order to provide a theoretical basis for the scientific management and rational utilization of forage grasses in the area.

## 2 Materials and methods

### 2.1 Overview of the study area

Ganzi River Plateau, located in the northeast sector of the Qinghai Lake, is an important grassland pastoral area in Haibei Tibetan Autonomous Prefecture. It comprises mainly mountain grassland and mountain meadow with an elevation of 3100-4360 m. The average annual temperature of the region is -3 °C, with maximum and minimum temperatures of 9 °C and -16 °C, respectively, and the average annual precipitation is 350-400 mm. The area lies within the semi-arid and alpine climatic zone of the plateau and the surface vegetation comprises predominantly by *Poa alpigena* L., *Stipa purpurea* Griseb., *Carex rigescens, Leymus secalinus, Polygonum sibiricum* Laxm. and *Allium przewalskianum*. In this study, the latitude and longitude of Poa rhizosphere soil and non-rhizosphere soil were 37°06’N; 100°31’E.

### 2.2 Sampling and treatment

Soil samples were randomly collected from four 2 m × 2 m quadrats, with each quadrat spaced more than 20 meters apart, using the S-type five-point sampling method. The rhizosphere soil was collected by the shaking root method and sieved by 2 mm sieve, whereas the non-rhizosphere soil was obtained from a 0-20 cm vertical soil profile within the projection range of the plant rhizosphere. Then soil samples from five points within a single soil quadrant were combined to form a composite sample. Four rhizosphere and four non-rhizosphere soil samples were individually placed in sterile bottles and marked as follows: GZG 1, GZG 2, GZG 3, and GZG 4 for the rhizosphere soil samples, and GZC 1, GZC 2, GZC 3, and GZC 4 non-rhizosphere soil samples. All samples were divided into two parts, one of which was placed in a 50 ml EP tube and immediately placed in liquid nitrogen for subsequent DNA extraction and meta-genomics sequencing. And the other part was used for the determination of soil physical and chemical properties.

### 2.3 Soil DNA extraction and library construction

Soil DNA extraction and purification were performed using the method described by Han et al. (2010). The crude DNA was extracted with chloroform-isoamyl alcohol, reprecipitated with isopropanol, and purified with QIAquick Gel Extraction Kit buffer. Having established DNA concentrations and integrity, BGI (Shenzhen, China) was entrusted to perform metagenomic high-throughput sequencing using BGISEQ-500 sequencing platform. The original number of microbial metagenomes and all data of metabolic pathways of each sample were screened by Linear discriminant analysis Effect Size (LEfSe) and enrichment analysis of microbial metabolic pathways was conducted (Zorrilla et al., 2021). The metagenomics sequencing data are available at the National Center for Biotechnology Information, USA (https://www.ncbi.nlm.nih.gov/sra/PRJNA867494). The accession numbers are SRX17159203, SRX17159204, SRX17159205, and SRX 17159206 for GZG group; and SRX 17159253, SRX17159254, SRX17159201, and SRX17159202 for GZC group.

### 2.4 Determination of soil physical and chemical properties

The soil moisture content was determined using JK-100F soil moisture meter (JINCHE, China) with an accuracy of 0.1%. soil carbon (TC) and nitrogen (TN) content were measure by CE-440 element analyzer (EAI., USA) and the Kjeldahl method. In addition, soil pH (1:2.5 soil/water suspension) and conductivity (1:5 soil/water leaching solution) were measured using PHS-25 acidity meter (LEICI, China) with an accuracy of 0.05 and DDS-307 conductivity meter (LEICI, China) with an accuracy of 0.01, respectively.

### 2.5 Data analysis

The raw data was sorted using the Trimmomatic software (v3.3) to remove joint sequences for high-quality valid sequences (Bolger et al., 2014). Based on default parameters (LEADING:3 TRAILING:3 SLIDINGWINDOW:4:15 HEADCROP:12 MINLEN:36), the cleaned linker sequences were as follows: PrefixPE/1: AAGTCGGAGGCCAAGCGGTCTTAGGAAGACAA; PrefixPE/2:AAGTCGGATCGTAGCCATGTCGTTCTGTGAGCCAAGGAGTTG.

The clean metagenome was assembled using MEGAHIT with “–min-contig-len 500 –preset meta-large”, and all sample sequences were combined for gene assembly. Metagenomic assembly was then evaluated using METAQUAST software and compared to the reference sequence to obtain information on the number of high qualities contigs, longest contig and N50 of the assembled sequence (Li et al., 2024). The sequences were compared and analyzed with the constructed species composition database using KRAKEN V2 software, whereas genomes including bacteria, archaea, fungi, viruses, protozoan genomes, nt database, etc. were downloaded from NCBI as the reference database. The species annotation and abundance were obtained by BRACKEN combined with taxonomic information database from NCBI, with data being classified at the phylum, class, order, family, genus, and species taxonomic levels. The microorganism Alpha diversity analysis was performed using VEGAN software in the R package and Beta diversity was analyzed based on principal co-ordinates analysis (PCoA) using R software.

Furthermore, indicator organisms and metabolic pathways were obtained based on LEfSe analysis of intergroup organism raw data and cumulative metabolic pathway data (Kyoto Encyclopedia of Genes and Genomes [KEGG] analysis), respectively (Segata et al., 2011). LEfSe analysis is employed to detect species that exhibit significant variation across two or more groups, potentially serving as biomarkers. The streamlined process is as follows: Initially, the Kruskal-Wallis rank sum test is applied to assess species abundance among groups, thereby identifying species with notable differences. Subsequently, the Wilcoxon rank sum test is conducted on the subspecies of the identified species to ascertain if they consistently align with a specific taxonomic rank. Ultimately, linear discriminant analysis (LDA) is utilized to pinpoint the definitive differential species.

The statistical analysis of the data was performed using SPSS 21.0 software. During Correlation analysis, p-values were adjusted for multiple comparisons using the False Discovery Rate (FDR) approach. Subsequently, the Benjamini-Hochberg (BH) method was applied to further refine these p-values. We filtered the corrected results for correlations with an absolute value of |r| > 0.8 and a p-value < 0.05. The visualization of these correlations was carried out with Cytoscape 3.10, where the sizes of the dots indicated the number of related genera. The more, the red and blue connecting lines indicate positive and negative correlations between two types of soil microorganisms, and the thickness of the lines represents the absolute value of the correlation between the two types of bacteria.

## 3 Results and analysis

### 3.1 Analysis of physicochemical properties between GZG and GZC groups

The total carbon, total nitrogen, water content, pH, and electrical conductivity of the soils were measured and shown in **Table 1**. It is evident that there are certain differences in the physical and chemical indicators of the soils between the GZG and GZC groups. Specifically, the water content, pH, total carbon, and total nitrogen content of the rhizosphere soil were significantly lower than that of the non-rhizosphere soil (*p*-Value < 0.05).

**Table 1:** Physicochemical properties of GZG and GZC.

Sample Site	Water content (%)	pH	Electrical conductivity (ms cm^−1^)	Total carbon (g kg^−1^)	Total nitrogen (g kg^−1^)
GZG	4.67 ± 0.35 b	7.88 ± 0.11 b	0.15 ± 0.03 a	3.06 ± 0.32 b	0.07 ± 0.01 b
GZC	5.14 ± 0.78 a	8.03 ± 0.13 a	0.15 ± 0.02 a	4.37 ± 0.38 a	0.20 ± 0.03 a

Values are means ± SD from four biological replicates, and statistically significant differences in soils are marked with different letters *(p* < 0.05).

### 3.2 Microbial composition of *P. alpigena* L. in the Ganzi River area

Each sample of metagenomic analysis was sequenced in excess of 10 GB, with an average sequence number of more than 36 million read pairs, indicating that the microorganisms were sequenced in sufficient quantities with good stability. A taxonomic study of the microbial populations showed that > 55% of the sequences from the samples were of unknown species (see **Table 2**). The percentage of known microbial sequences in the soil of the non-rhizosphere sample was 37.42%-39.19%, and the percentage of known microbial sequences in the rhizosphere was 36.35%, which was slightly lower than that of the non-rhizosphere sample.

**Table 2:** Microbial sequencing results of *P. alpigena* L. soil in Ganzi River area.

Project	Nature (GZC)	Rhizosphere soil (GZG)
Number of clean reads (a couple)	36166724.5 ± 2289612.7 a	35997544.5 ± 1962755.1 b
Classified reads (%)	44.45 ± 0.75 a	42.54 ± 0.66 b
Chordate reads (%)	6.17 ± 0.07 a	6.04 ± 0.11 a
Unclassified reads (%)	55.56 ± 0.75 b	57.47 ± 0.66 a
Microbial reads (%)	38.14 ± 0.80 a	36.35 ± 0.76 b
Bacterial reads (%)	30.56 ± 0.75 a	28.96 ± 0.81 a
Viral reads (%)	0.15 ± 0.00 a	0.15 ± 0.00 a
Fungal reads (%)	1.40 ± 0.02 a	1.34 ± 0.03 b
Protozoan reads (%)	0.18 ± 0.00 a	0.18 ± 0.01 a

The percentage in the table refers to the percentage of corresponding sequences in the total sequence. The data is represented by mean ± SD from four biological replicates, and the significance of different soils is represented by different letters (p Value<0.05).

Analysis of the relative abundance of the dominant microorganisms in all samples showed that there were high levels of duplication of microorganisms at different taxonomic levels. According to the analyzed microbial data, bacteria were the most dominant in the samples, accounting for > 98% of all soil samples. This was followed by archaea, with an average of 0.72% and 0.59% in rhizosphere and non-rhizosphere soils, respectively, and finally by eukaryotes and viruses.

**Figure 1:**
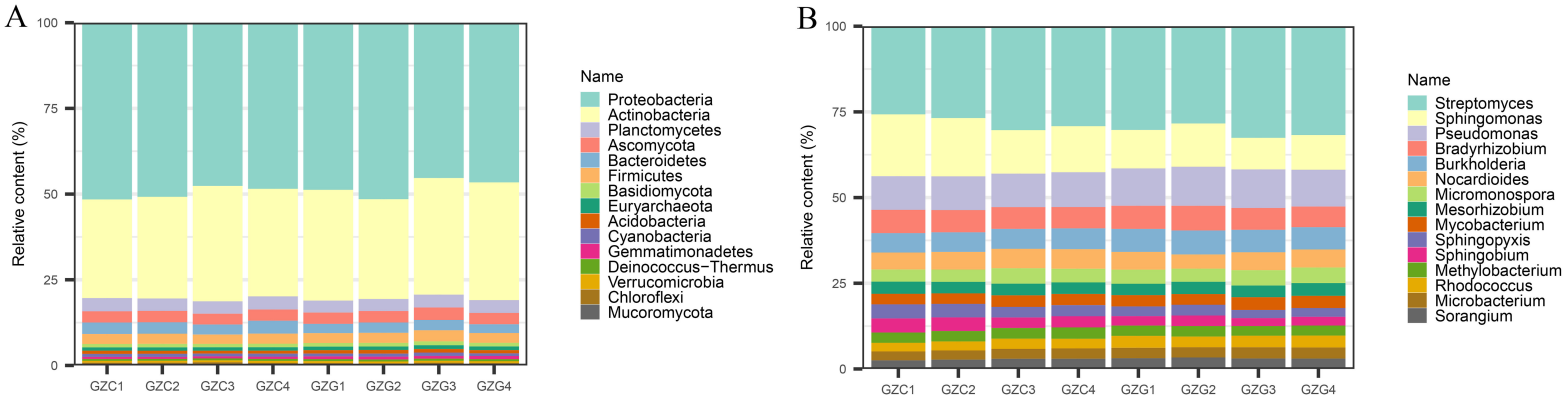
The relative abundances of microbial at phylum **(A)** and genera **(B)** in GZG and GZC.

The cleaned sequences of each sample were analyzed against databases using KRAKEN 2 and BRACKEN to obtain information regarding the content of various microorganisms at different taxonomic levels. A total of 61 phyla were detected, with the highest being Proteobacteria, with an average of 36.54% and 35.09% in the GZC and GZG groups, respectively as shown in **Figure 1A**. This was followed by Actinobacteria, which accounted for between 21.15 and 25.25% of the total. A total of 1,606 genera were detected, and the highest abundance in all samples was of the genus *Streptomyces*, with an average of 6.16% and 6.43% in the GZC and GZG groups, respectively. This was followed by *Sphingomonas* spp. and *Pseudomonas* spp. These differences in content reached statistical significance (*p*-Value < 0.05) and highly significant levels (*p*-Value < 0.01) in the GZC and GZG groups respectively (see **Figure 1B**).

**Figure 2:**
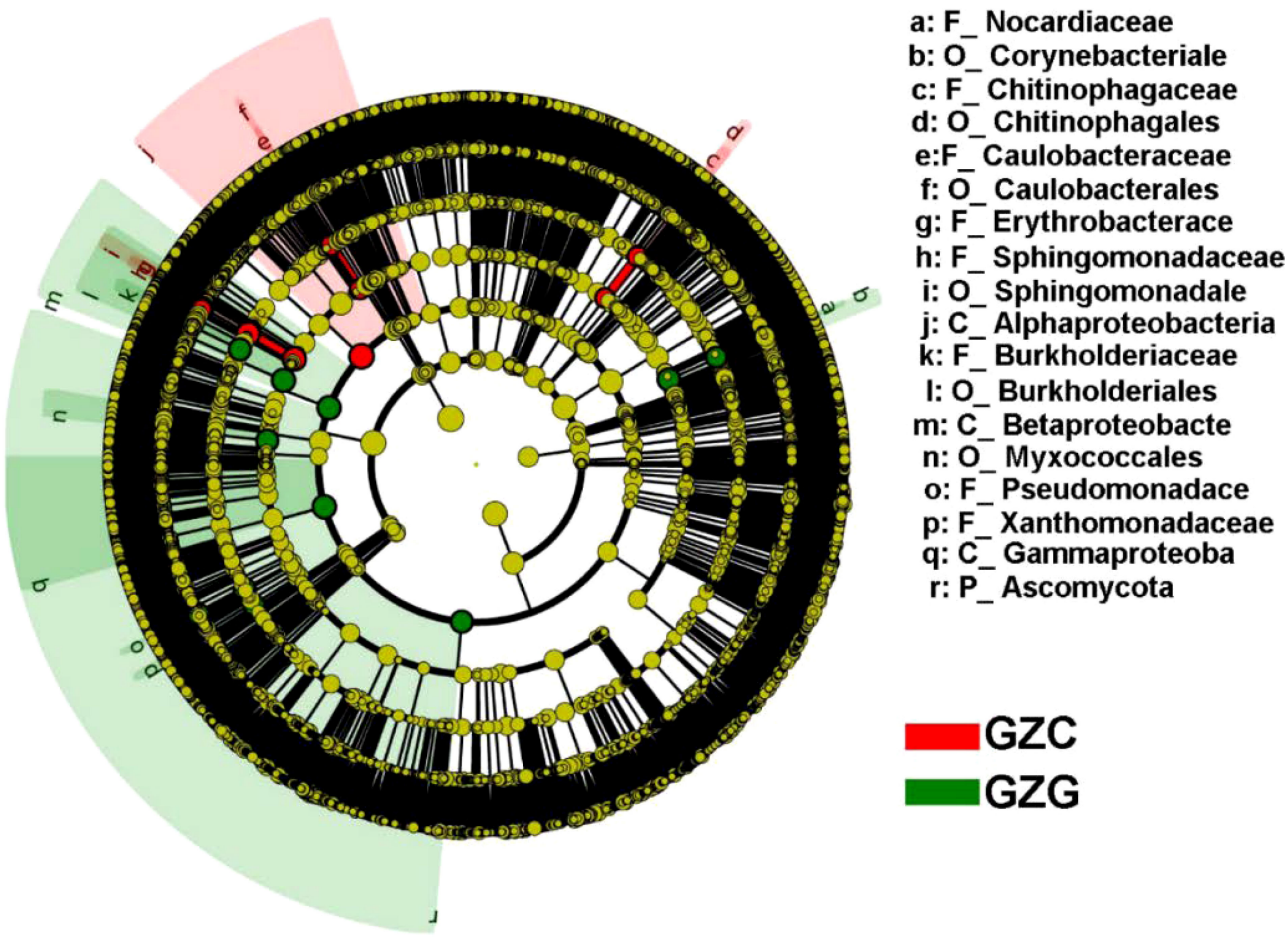
LEfSe analysis of GZG and GZC microorganisms.

We further classified the different microorganisms in rhizosphere and non-rhizosphere soils. The results showed that the level of differences ranged across phylum to family. For example, the GZG group had significantly higher microbial abundance in terms of Ascomycota, Pseudomonadace, and Burkholderiaceae than the GZC group, while the GZC group had significantly higher microbial abundance of Alphaproteobacteria and Caulobacterales than the GZG group, as seen in **Figure 2**.

The circular layers delineate the taxonomic hierarchy, progressing from the center outwards as follows: Kingdom, Phylum, Class, Order, Family, Genus, and Species. Node size corresponds to the species’ abundance, where yellow indicates no significant variation. Red and green nodes denote key microbial groups within the GZG and GZC groups, respectively. Differential species are marked with letters, with the corresponding species detailed in the legend on the right side. Due to space constraints, the legend on the right is limited to species from the Phylum to Genus levels.

### 3.3 Microbial diversity of *P. alpigena* L. in the Ganzi River area

**Figure 3:**
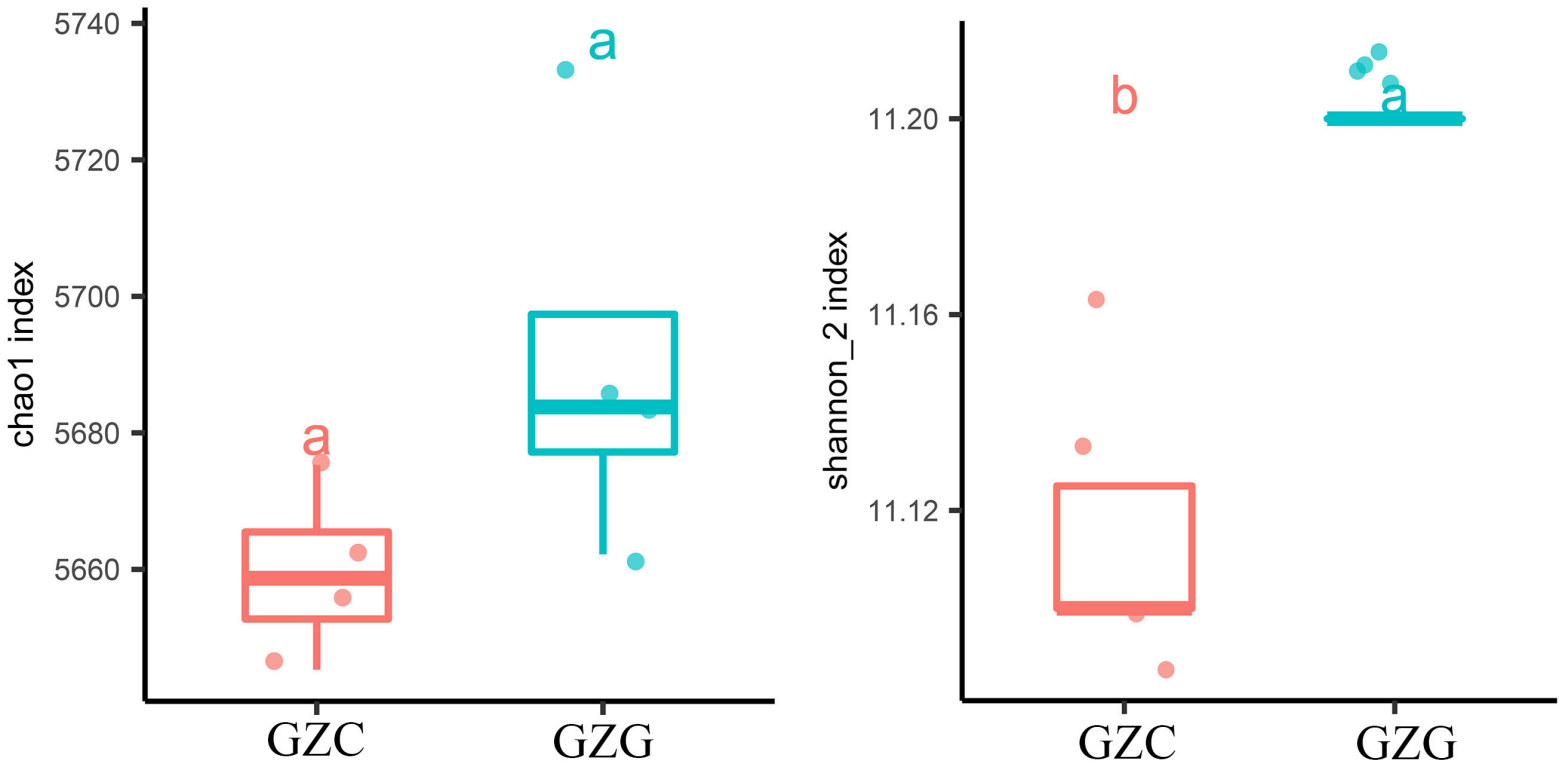
α-diversity box plot. Left: Chao 1 index. Right: Shannon index. Different lowercase letters indicate significant differences (p< 0.05).

The inter- and intra-species diversity and richness of microorganisms in the samples can be obtained by the analysis of the Chao 1 index and Shannon index in **Figure 3** (Sun et al., 2021b). Chao 1 is commonly used in ecology to estimate the total number of species and to assess the index of microbial abundance in the community. The results showed that the Chao 1 index was lower in the GZC group (*p*-Value < 0.05). Shannon index is a measure of species diversity, with higher values indicating greater diversity. In **Figure 3**, with a base of 2, the Shannon index values for GZG are consistently higher than those for GZC. These indicated a significantly greater richness and diversity value for GZG microorganisms compared to GZC.

**Figure 4:**
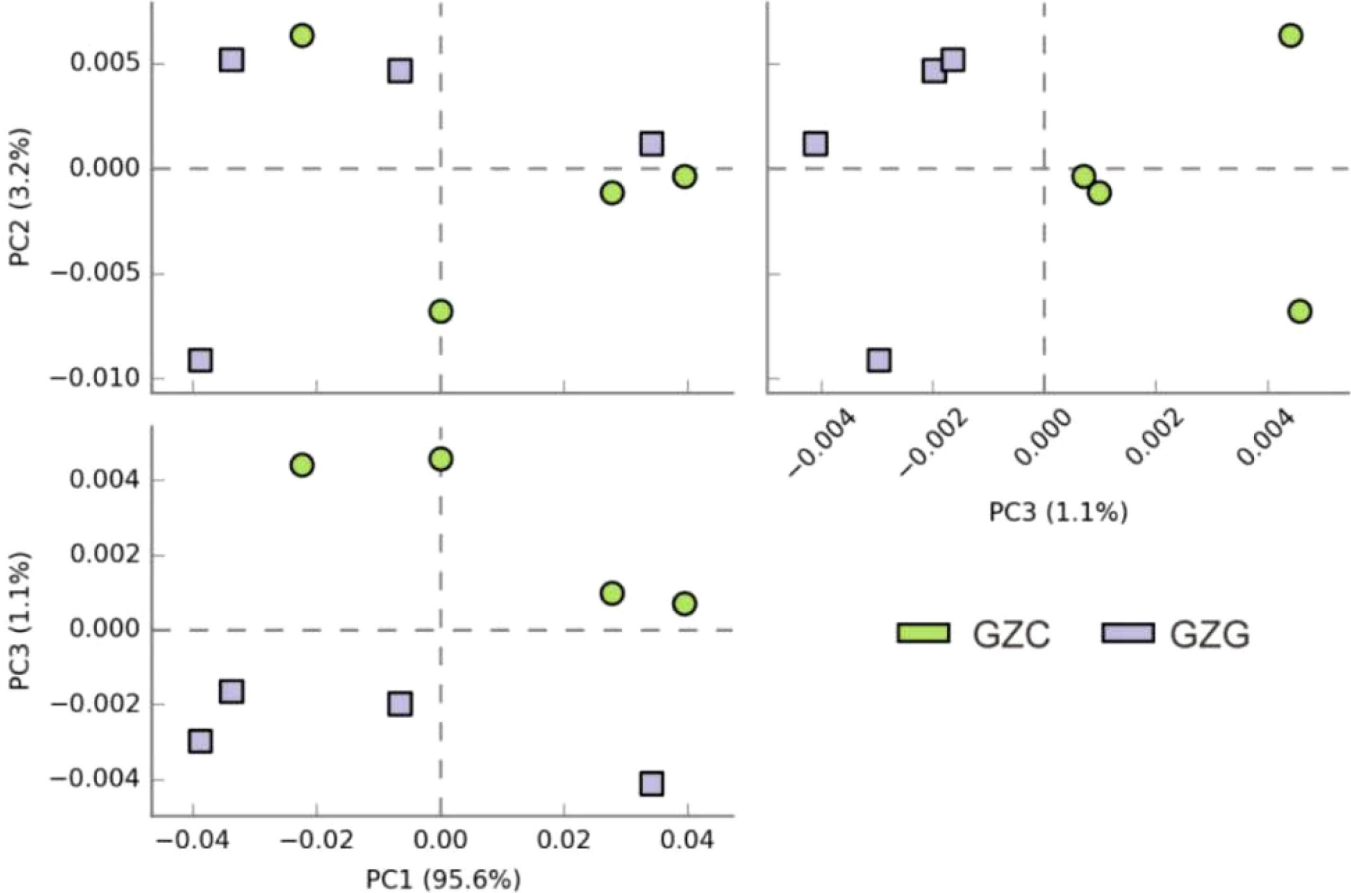
Results of PCA analysis biplot of GZG and GZC microorganisms.

Principal Component Analysis (PCA) is a standard approach for discerning variations across datasets, converting the original data into a series of linearly independent components via linear transformation. **Figure 4** depicts the disparities between sample groups through the distances between individual sample points. At the genus level, the first three principal components (PC1, PC2, and PC3) account for a cumulative explanation rate of 99.9%. While the scatter points are spread out, suggesting pronounced differences between groups, the distinctions among non-rhizosphere microbes are subtler. The findings indicate that the GZC communities exhibit a high degree of similarity in their composition, in contrast to the GZG communities, which show a degree of variability in their distribution.

### 3.4 Differences in microbial metabolic pathways between GZG and GZC groups

**Figure 5:**
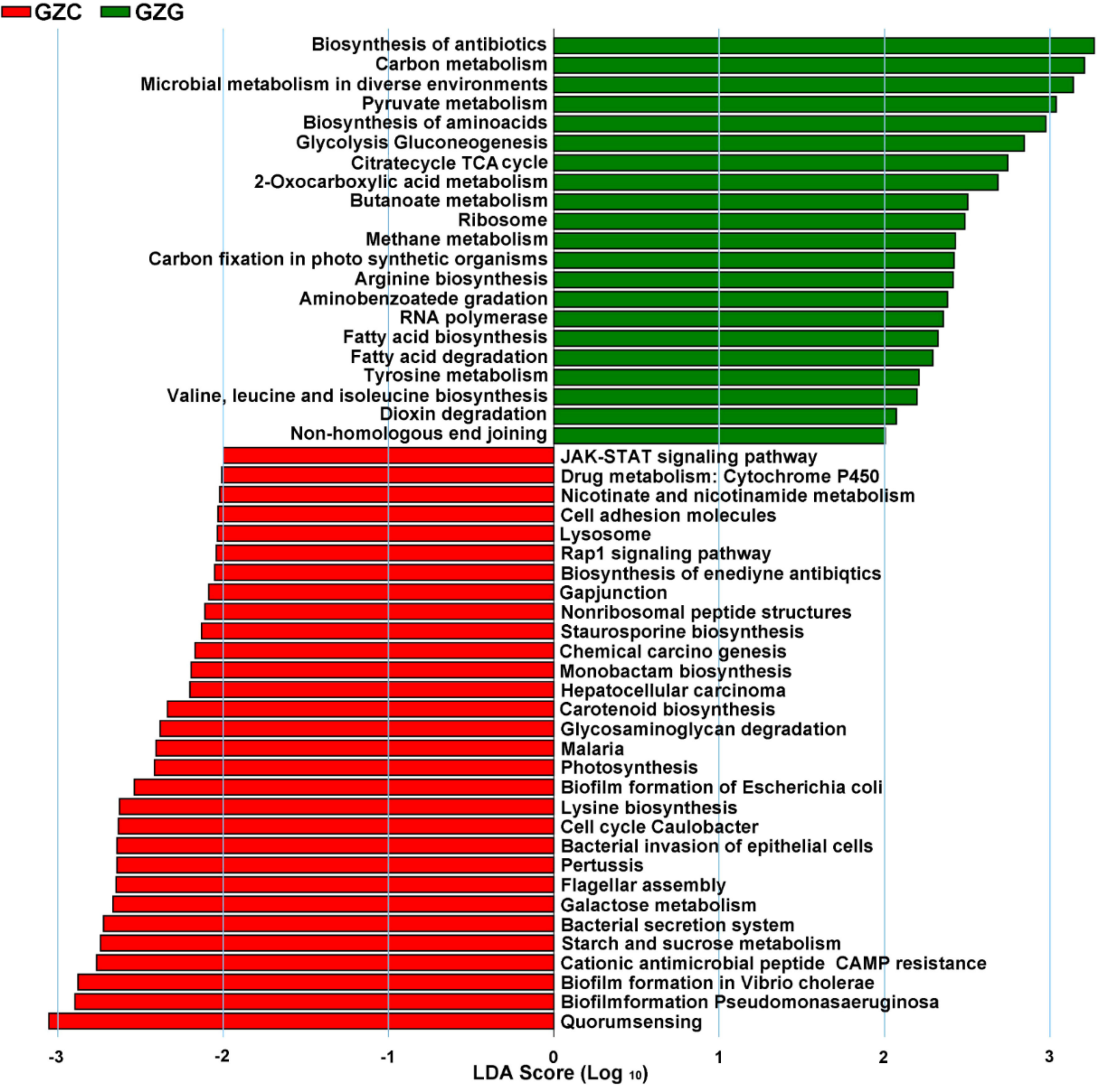
The soil microbial metabolic pathways by LEfSe analysis.

The raw counts data for each gene obtained by Salmon software was used to count the number of genes in each metabolic pathway. Information regarding key metabolic pathways of rhizospheric and non-rhizospheric microorganisms was obtained using LEfSe software. The results showed that a total of 51 differential metabolic pathways were obtained, as shown in **Figure 5**. Among them, there were 21 and 30 differential metabolic pathways in the GZG and GZC groups, respectively. The pathways significantly enriched in the GZG group mainly involved biosynthesis of antibiotics, carbon metabolism, methane metabolism, aminobenzoate degradation, RNA polymerase, fatty acid, fatty acid biosynthesis/degradation, and microbial metabolism. RNA polymerase, fatty acid, fatty acid biosynthesis/degradation, microbial metabolism in diverse environments, and carbon fixation in photosynthetic organisms; see **Figure 5**.

Pathways significantly enriched in the GZC group mainly involved quorum sensing, staurosporine biosynthesis, starch and sucrose metabolism, nicotinate and nicot in amide metabolism, monobactam biosynthesis, lysosome, glycosaminoglycan degradation, gap junction, drug metabolizing cytochrome P450, chemical carcinogenesis, and cell adhesion molecule pathways. The pathways of rhizosphere and natural soil microbial enrichment differed significantly, indicating that rhizospheric microorganisms altered their original metabolic pathways.

### 3.5 Correlation analysis of rhizospheric and non-rhizospheric microorganism

**Figure 6:**
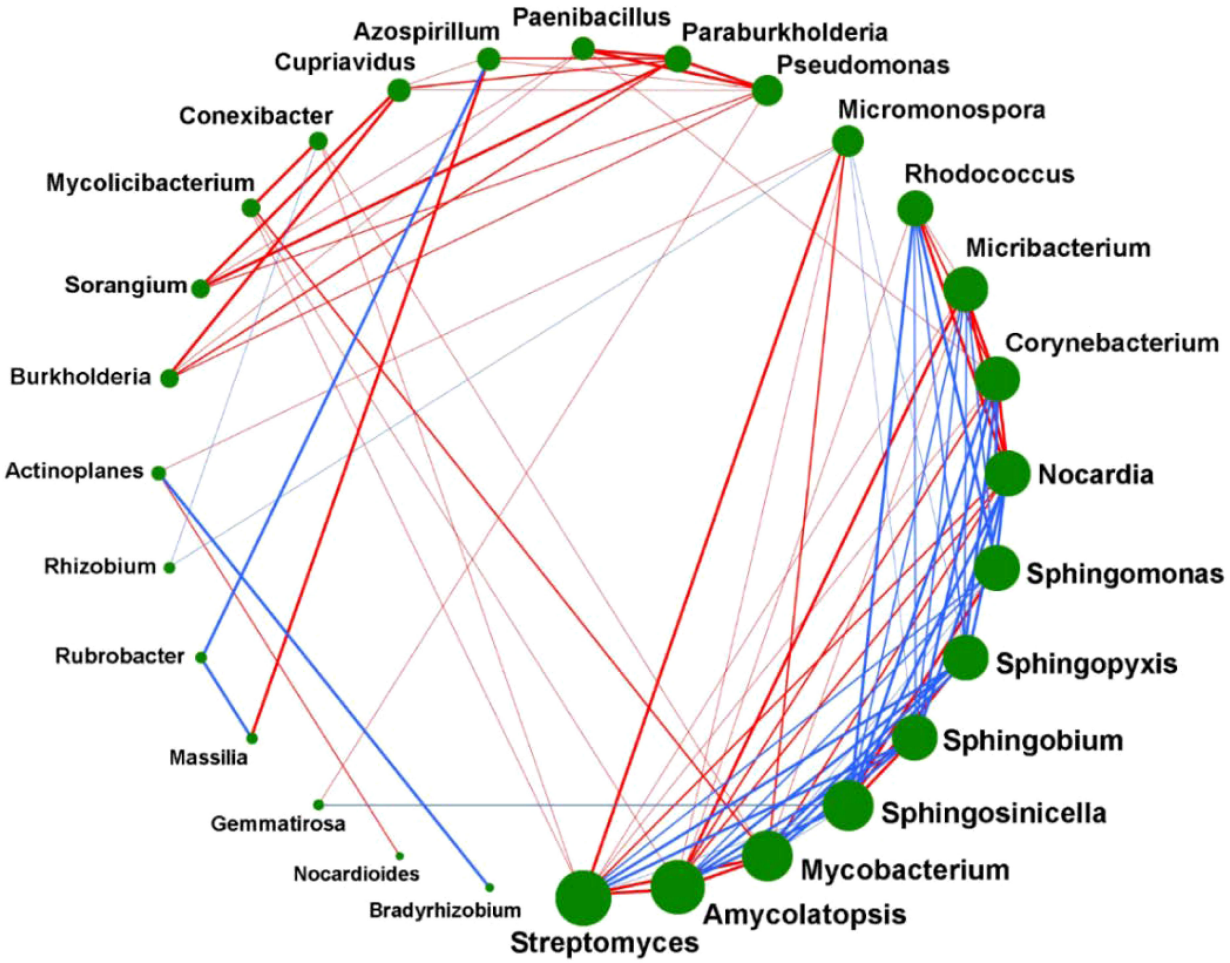
Correlation analysis between rhizosphere and non-rhizosphere soil microorganisms.

In order to study the correlation between the abundance of different microorganisms in the Ganzi River area, we conducted a correlation analysis of the top 28 microorganisms in the GZC and GZG groups. As seen in **Figure 6**, green dots represent different microorganisms, the sizes of the dots indicated the number of related genera. The more, the red and blue connecting lines indicate positive and negative correlations between two types of soil microorganisms, and the thickness of the lines represents the absolute value of the correlation between the two types of microorganisms. The results showed that the top three groups of bacteria with the highest content were *Streptomyces*, *Amycolatopsis* and *Mycobacterium*, the next four groups were *Sphingosinicella*, *Sphingobium*, *Sphingopyxis and Sphingmonas.* These highly abundant microorganisms play a key role in environmental remediation and biodegradation, corresponding to the anthropogenic development and pollution of the Gan River. The abundance of different microorganisms exhibited significant positive or negative correlations (*p*-Value < 0.05), indicating that different microorganisms interacted closely and jointly influenced metabolic processes in the soil.

## 4 Discussion

### 4.1 Differences in abundance of dominant microorganisms between rhizosphere and non-rhizosphere soils

In this study, we used metagenomic techniques to identify and classify a variety of microorganisms and counted the proportion of each microorganism community in the total population. The results showed that at the phylum level, the highest relative abundance in both rhizosphere and non-rhizosphere soils was the phylum Proteobacteria, followed by the phylum Actinobacteria. Their total contents reached nearly 80% and formed a majority, which is consistent with the results of previous studies. For example, the dominant soil bacteria in alpine meadows belonged to the phyla Proteobacteria, Acidobacteria and Actinobacteria (Cong et al., 2022). Zhao et al. (2022) used Illumina MiSeq second-generation high-throughput sequencing technology to study bacterial communities of different degraded grasslands in the eastern Qilian Mountains and found that the dominant bacteria were Proteobacteria and Actinobacteria. Zhang et al. (2008) studied microbial diversity in mangrove silts and found that the majority were Proteobacteria. Proteus and Actinobacteria were the dominant species in the Ganzi River area of Qinghai Lake, which indicates that the species abundance at the phylum level of soil microorganisms is less influenced by rhizosphere or non-rhizosphere soils. The phylum Proteobacteria is the most dominant in most soil habitats. This phenomenon suggested that population adaptation by Proteobacteria can be considered as r-selective, i.e., having a high reproductive capacity and being more adaptable in unstable environments compared to other species (Baquero et al., 2021). This means that when microorganisms of the Proteobacteria phylum enter a new environment or when a plant enters that soil environment, microorganisms of this population respond and are able to attach themselves near the plant roots in a relatively short period of time, thus dominating the rhizosphere ecosystem (Zhu et al., 2016).

Stamp-based analysis of rhizospheric and non-rhizospheric microorganisms showed that the abundance of some microbial species differs significantly in different environments. For example, the abundance of Gemmatimonadetes in the GZG group was significantly higher (*p*-Value < 0.01) compared to the GZC group (see **Supplementary Figure S1**). Research shows that this type of microorganism has a strong nitrogen removal effect, thus the abundance of these microorganisms was negatively correlated with nitrogen content and was more adapted to drier environments (DeBruyn et al., 2011). In this study, the water and nitrogen contents of rhizosphere soil in this area were significantly lower than that of non-rhizosphere soil, so Gemmatimonadetes was more suited to the rhizosphere soil environment (Shang et al., 2022).

### 4.2 Differences in microbial metabolic pathways between rhizosphere and non-rhizosphere soil

Studies have shown that soil microorganisms in different environments differ in the metabolic pathways in which they participate, and that soil microorganisms are constantly adapting to environmental changes (Palit et al., 2022). In this study, we found that metabolic pathways of rhizospheric and non-rhizospheric microorganisms were significantly enriched in carbon metabolism, tricarboxylic acid cycle (TCA), aminobenzoate degradation and methane metabolism, in addition to certain metabolic pathways related to usual life activities. Carbon metabolism and TCA metabolism can provide energy required for biological life activities and supply various precursors for other life activities, indicating that rhizospheric microorganisms are metabolically active (Ofaim et al., 2017). Aminobenzoate is an important raw material and a limiting factor in tryptophan synthesis. It is a precursor substance for plant hormone synthesis, and enrichment of this pathway indicates that rhizospheric microorganisms have attained a strong symbiotic relationship with plants (Liu et al., 2015). The significant enrichment of methane metabolism indicated the enhanced ability of rhizospheric microorganisms of *P. alpigena* L. to metabolize methane in soil, thus providing an important pathway to mitigate and control elevated methane levels and suppress global warming (Che et al., 2022).

Pathways such as quorum sensing, formation of *Pseudomonas aeruginosa* biofilms and cytochrome P450-dependent drug metabolism were significantly enriched in non-rhizospheric microorganisms. Among them, the quorum sensing system is a physiological effect that regulates gene expression based on the density of surrounding microorganisms (Li et al., 2023). A weakened effect indicates a decrease in the content of surrounding microorganisms, which indirectly shows that the plant root system has a strong selection effect on microorganisms; if detached from the root system, the effect of limiting microorganism reproduction will be relatively weakened. It is known that the quorum sensing system is one of the important ways of information exchange between plant roots and microorganisms and is also an effective mechanism for intra- and inter-species information transfer by microorganisms (Majdura et al., 2023). As it is selected by the plant rhizosphere system, the quorum sensing mechanism will be weakened accordingly, which is consistent with the results of this paper. *P. aeruginosa* has strong antimicrobial resistance and can weaken host resistance. Enrichment of this pathway indicates that the inhibition of harmful bacteria is weakened in the non-rhizosphere environment, while the roots of *P. alpigena* L. may have the ability to inhibit *P. aeruginosa* reproduction (Tuon et al., 2022). Cytochrome P450 participates in the metabolism of endogenous and exogenous chemicals containing drugs and active compounds. It is an important enzyme in the metabolism pathways, has an important role in the regulation of cytokines, and can remediate contaminated environments (Minerdi et al., 2023).

### 4.3 Correlation analysis among soil microorganisms of *P. alpigena* L.

Soil microorganisms are comprised of a variety of different microbial groups. It is inevitable and reasonable that all microbial groups can form mutually promoting or inhibiting relationships with each other. This competition is usually for energy and resources, and is known as ecological niche differentiation, with the ultimate goal of maximizing the ecological fitness of populations by means of competition or symbiosis (Wang et al., 2021). The seven most abundant microorganisms play a crucial role in environmental remediation and biodegradation, and there is a positive correlation among them, which corresponds to the development and anthropogenic pollution of the Ganzi River (Dou et al., 2021).

The correlation analysis of varying microorganism contents revealed that the presence of Burkholderia spp. enhanced the levels of Sorangium spp. and Cupriavidus spp., respectively. These three microorganisms are interconnected by a bold red line, signifying their strong association. This is because *Burkholderia* spp. is an effective microorganism in promoting plant growth and adapting to the environment, while Sorangium spp. will provide many metabolites that are beneficial to plant growth (Sun et al., 2021a). At the same time, it has been shown that *Cupriavidus* spp. can play a role in the non-rhizosphere of bacterial diseases of crops and participate in the regulatory mechanism of heavy metal resistance. These three bacteria play complementary roles which have certain effects on the growth of plants. This conclusion is consistent with that of Elshafie and Camele (2021) in that Burkholderia spp. are beneficial to plants and can promote the colonization of some beneficial bacteria, so it is reasonable to consider that there is a certain promotional relationship between the rhizospheric microorganisms of plants.

*Micromonospora* is commonly found in the water and mud of lakes and rivers. It is able to break down some organic matter that is not easily decomposed, such as cellulose, chitin, and xylan. A variety of antibiotics are produced, especially aminoglycosides, such as gentamicin, which is effective against *P. aeruginosa* (Kaewkla et al., 2021). Streptomyces spp. is a taxon that produces important antibiotics and is an important medicinal resource. This genus is widely distributed in soils with high salinity and in extreme environments (Sivalingam et al., 2019). Our research shown a positive correlation between the abundance of *Micromonospora* and Streptomyces spp. in the Ganzi River area, which is conducive to improving and enhancing the root development of *P. alpigena* L. as well as its adaptability to external environmental stresses, providing support for environmental restoration in the Qinghai Lake basin. In addition, Schütze et al. found that actinomycetes synthesize diverse antibiotics to secure carbon sources, inhibiting microbes that impede their acquisition and thus maintaining their competitive edge (Schütze et al., 2014).

## 5 Conclusion

The species richness of non-rhizospheric microorganisms of *P. alpigena* L. in the Ganzi River area was higher than that of rhizospheric soil microorganisms. The percentage of dominant microorganisms in the two soils had small differences, and the phylum Proteobacteria had the largest percentage in the two types of soils, whereas the roots of *P. alpigena* L. were more selective of the microorganisms in the soil environment. The results of Kyoto Encyclopedia of Genes and Genomes (KEGG) enrichment analysis showed that rhizospheric microorganisms were significantly enriched in pathways involving biosynthesis of antibiotics, carbon metabolism, methane metabolism, and aminobenzoate degradation, while pathways of non-rhizospheric microorganisms were dominated by quorum sensing, lysosomes, and the drug metabolizing cytochrome P450. Correlation analysis of the abundance of dominant microorganisms in all soil samples revealed a mutual inhibitory or promotional relationship between different species of microorganisms.

The Qinghai Lake basin stands as a sentinel of ecological security in the northeastern Qinghai-Tibet Plateau and a natural buffer against the advancing desertification from the west. This region, with its distinctive ecosystems, harbors abundant soil microbial resources that are vital for the sustenance of ecological functions. Our study aims to create a thorough database of these soil microbial resources for the grasslands of the Ganzi River Nature Reserve, providing a scientific basis for the conservation and restoration of these critical habitats, and thereby supporting sustainable development in the wider Qinghai Lake area.

## Data Availability

The datasets presented in this study can be found in online repositories. The names of the repository/repositories and accession number(s) can be found below: https://www.ncbi.nlm.nih.gov/, PRJNA867494.

## Supplementary material

Supplementary Figure 1 Scale plot of characteristic sequence of Gemmatimonadetes.
