# Supplementary figures and images for: Metagenomics-based study of rhizospheric microorganisms of Poa alpigena L. in Qinghai Lake, Ganzi River Plateau
Source: Front Plant Sci. 2025 Feb 21;15:1518637. doi: 10.3389/fpls.2024.1518637 (PMC11885292; doi:10.3389/fpls.2024.1518637)

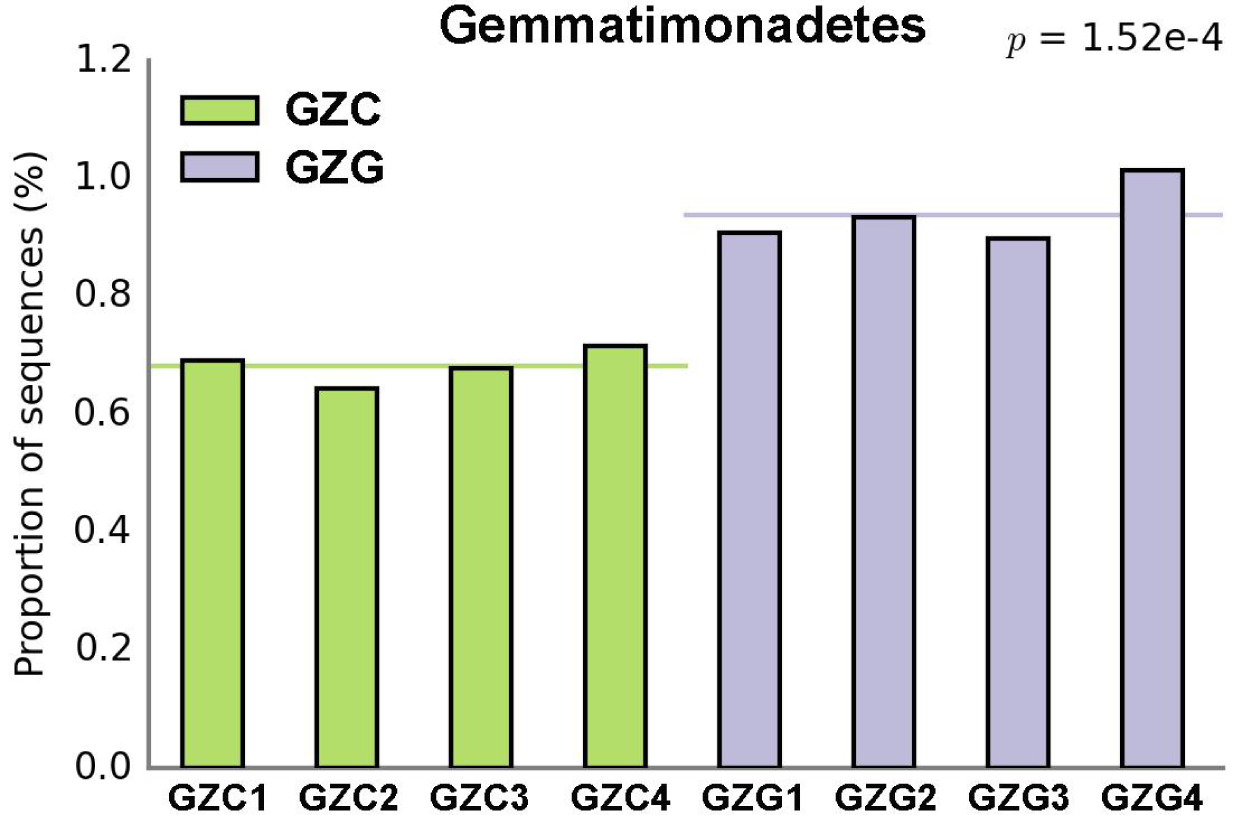

Supplement: Supplementary Figure 1 — Scale plot of characteristic sequence of Gemmatimonadetes. [file Image1.tiff]
